# New Insights into Psoriasis

**DOI:** 10.3390/ijms232112851

**Published:** 2022-10-25

**Authors:** Boguslaw Nedoszytko, Agnieszka Owczarczyk-Saczonek, Dorota Krasowska, Aneta Szczerkowska-Dobosz

**Affiliations:** 1 Department of Dermatology, Venerology and Allergology, Medical University of Gdańsk, 80-211 Gdańsk, Poland; 2 Molecular Laboratory, Invicta Fertility and Reproductive Center, 81-740 Sopot, Poland; 3 Dermatology, Sexually Transmitted Diseases and Clinical Immunology, School of Medicine, University Warmia and Mazury in Olsztyn, 10-719 Olsztyn, Poland; 4 Department of Dermatology, Venereology and Pediatric Dermatology, Medical University of Lublin, 20-081 Lublin, Poland

Psoriasis is a chronic inflammatory skin disease with many comorbidities resulting from not only local but also systemic inflammation. The pathogenesis of the disease is complex and not fully understood. From a genetic point of view, psoriasis is nowadays classified as a multifactorial disease. The interaction (epistasis) of multiple genes from different loci, epigenetic phenomena regulating gene expression and environmental and infectious factors inducing the onset of disease symptoms participates in its complex pathogenesis [1,2]

The current Special Issue of *IJMS*, titled “New Insights into Psoriasis,” presents four original and five review articles on the pathogenesis and modern treatments of psoriasis.

Two articles focused on the role of vitamin D in psoriasis [3,4]. Brożyna et al. [3] presented the role of vitamin D and its active metabolites in the pathogenesis, the modulation of the local neuroendocrine system and treatment of psoriasis. Wierzbicka et al. [4] studied the effect of vitamin D on the expression of IL-33 and its receptor ST2 in keratinocytes, melanocytes, fibroblasts, and basal cell carcinoma cells in vitro. It was shown by the authors that 1,25(OH)2D3 effectively stimulates the expression of IL-33 and its receptor ST2’s mRNAs in a time-dependent manner, in keratinocytes and to the lesser extends in melanocytes, but not in fibroblasts. The authors concluded that that vitamin D can modulate IL-33 signaling, opening up new perspectives for our understanding of the mechanism of vitamin D action in psoriasis therapy.

Marek-Jozefowicz et al. [5] describe the role of deregulation of brain–skin axis in psoriasis. The pathophysiological mechanisms suggest a role for nerve-related factors, namely, their interaction with mast cells and the severity of neurogenic inflammation in this regard, and deregulation of the crosstalk between endocrine, paracrine, and autocrine stress signaling pathways. Stress redistribution with increased transport of leukocytes into the skin can exacerbate psoriasis. The severity of psoriatic lesions contributes to the self-isolation of patients and the development of depressive disorders in some of them patients.

A new aspect of pathogenesis of psoriasis is indicated in article of Alalaiwe et al. [6] The authors studied the differentiated THP1 cells, stimulated by imiquimod (IMQ), which were utilized as the activated macrophage model. IMQ was also employed to produce psoriasis-like lesions in mice. A transcriptomic assay of macrophages revealed that the expressions of pro-inflammatory mediators and GDAP1L1 were largely increased after an IMQ intervention. The depletion of GDAP1L1 by short hairpin (sh)RNA could inhibit cytokine release by macrophages. Besides GDAP1L1, another mitochondrial fission factor, Drp1, translated from the cytosol to mitochondria after IMQ stimulation, followed by the mitochondrial fragmentation. After depleting GDAP1L1, the THP1 recruitment in the lymph nodes was decreased. The skin histology showed that the GDAP1L1-mediated macrophage activation induced neutrophil chemotaxis and keratinocyte hyperproliferation. The authors concluded that mitochondrial fission can be a target for fighting against psoriatic inflammation.

Czerwińska et al. [7], in a review study, analyzed original papers presenting significant research findings which may help to understand and interpret the NET (neutrophil extracellular trap) formation process in psoriasis. The protective activity of NETs based on the direct contact of trapped microorganisms with bactericidal components such as histones, granules released via degranulation and negatively charged DNA has been widely reported. Moreover, NETs promote relief from inflammation and wound healing by accumulating inflammatory mediators, and by degrading pro-inflammatory cytokines and chemokines. However, the course of psoriasis is characterized by a high accumulation of neutrophils both in plaques and blood, and a decrease in circulating neutrophils is accompanied by the regression of psoriatic plaques. The participation of neutrophils in the pathogenesis of disease is dependent on an autoinflammatory feedback loop between neutrophils, lymphocytes, dendritic cells and keratinocytes. Thus, the influence of NETs is observed during every step of the development of psoriasis. The enhanced expression of NETs causes the intensive secretion of both IL-17 and inflammatory mediators, which consequently results in a multiplied amount of neutrophils. The results of recent studies identified a combined influence of NETs and genetic conditions in Th17 induction. Regrettably, knowledge of the neutrophil mechanism in psoriasis is still incomplete and further studies are needed to develop new therapies directed at the NET formation process without damaging neutrophils.

Nowowiejska et al. [8], based on Pubmed database, focuses on a variety of aberrations in lipids in the skin, blood, and adipose tissue in psoriatic patients and their multifactorial impact on the pathogenesis of the disease. Lipids play a key role in psoriasis. Psoriatic patients suffer more often from hyperlipidemia and are prone to develop metabolic syndrome, atherosclerosis, and thus cardiovascular disorders. The authors discuss in details association of psoriasis with metabolic disorders, aberrations in lipid expression and their metabolism in psoriatic patients, the influence of lipid-lowering drugs on psoriasis and the impact of systemic antipsoriatic agents on lipidemia.

Psoriasis is frequently accompanied with metabolic syndrome or its components. The mechanism how obesity and dyslipidemia contribute to the pathogenesis of psoriasis is not fully known. To investigate the mechanisms by which obesity and dyslipidemia exacerbate psoriasis, Ikeda et al. [9] used wild-type and *Apoe*-deficient dyslipidemic mice, and administered a high-fat diet for 10 weeks to induce obesity. Imiquimod was applied to the ear for 5 days to induce psoriatic dermatitis. To examine the innate immune responses of NHEKs authors cultured and stimulated NHEKs using IL-17A, TNF-α, palmitic acid, and leptin. The authors revealed that obesity and dyslipidemia synergistically aggravated psoriatic lesions associated with increased gene expression of proinflammatory cytokines and chemokines and it was shown that metabolic-disorder-associated inflammatory factors, palmitic acid, and leptin augment the activation of epidermal keratinocytes.

Purzycka-Bohdan et al. [10], in the review entitled “Analysis of the Potential Genetic Links between Psoriasis and Cardiovascular Risk Factors”, presented the current state of knowledge on the potential genetic background of psoriasis and concomitant disorders that increase the risk of cardiovascular diseases (CVDs), such as hypertension, diabetes, dyslipidemia, obesity, metabolic syndrome and depression [8]. There is mounting evidence that the co-occurrence of psoriasis and risk factors of CVDs may originate from the pleiotropic mechanisms of interactions with many genetic pathways. The authors pointed to genes that may be involved in the development of both diseases and highlighted that understanding of the processes linking psoriasis with CVD may lead to improvement of psoriasis management in the future. Moreover, the authors listed the possible clinical implications and benefits from the future genetic studies on psoriasis and risk factors of CVDs including early patients’ screening and education leading to prophylaxis and treatment, primary disease control and better life quality, assessment of the response to treatment and decrease in cardiovascular risk as well as decrease in mortality rate.

Mercel Šabović et al. [11], in a review article, present the common pathophysiological mechanisms of the psoriasis and cardiometabolic disorders and the atherosclerotic impact of some psoriasis treatments. The authors point out future perspectives in the treatment of psoriasis that should be accompanied by medications that target psoriasis-driven atherosclerosis. This idea is based on shared pathways, including endothelial dysfunction, oxidative stress, arterial and cardiac remodeling, increased angiogenesis, prothrombotic state, monocyte and neutrophil recruitment and T cell activation. Furthermore, more studies should also assess the impact of adjuvant cardiovascular prevention agents, particularly statins, metformin, SGLT-2 inhibitors, and GLP-1 receptor agonists, on primary endpoints of psoriasis and surrogate markers of atherosclerotic cardiovascular disease. Moreover, the authors suggest that the inflammatory status of patients should be evaluated prior to starting antipsoriatic treatment and preventive measures of atherosclerosis as the most frequent course of morbidity and mortality in psoriatic patients should be implemented.
